# Cognitive Decline, Financial Decision-Making, and Wealth of Older Households

**DOI:** 10.1093/geroni/igaf122.1439

**Published:** 2025-12-31

**Authors:** Rashmita Basu

**Affiliations:** East Carolina University, Greenville, North Carolina, United States

## Abstract

Cognitive ability can impact older adults’ economic well-being. This study investigated the relationship between cognitive decline and switching financial responsibility within the household and its impacts on household wealth using the Health and Retirement Study (HRS) from 2006-2020. Cognitive decline and financial responsibility were measured for each HRS respondent aged 50 years or older. A decrease in objective test scores measured cognitive decline. Two primary outcomes included switching financial responsibility within the household and financial wealth. The analysis focused on the extent to which individuals’ cognitive decline was related to switching the financial responsibility within the household and if there was any impact on changes in financial wealth due to cognitive decline. Between two consecutive waves from 2006 to 2020, approximately 35% of respondents experienced cognitive decline, 35% improved, and 15% received the same cognitive score. Thirty-five percent did not switch to the role of financial responsibility within the household. Compared to those with no change in cognitive scores, those who experienced improvement or decreased scores were 26% and 27% more likely not to switch the financial responsibility role within the household. Further analysis examined if self-reported memory changes versus the objective value of cognitive scores influenced the switching decision within the household and its impacts on household wealth over time. This study provides an expanded profile of the variation in individual characteristics of a financial respondent within the household that leads to the switching decision of the financial responsibility role and its impact on household wealth.

